# Crystal structure of the [2Fe–2S] protein I (Shethna protein I) from *Azotobacter vinelandii*

**DOI:** 10.1107/S2053230X21009936

**Published:** 2021-10-19

**Authors:** Burak V. Kabasakal, Charles A. R. Cotton, James W. Murray

**Affiliations:** a Department of Life Sciences, Imperial College London, Exhibition Road, London SW7 2AZ, United Kingdom; b Turkish Accelerator and Radiation Laboratory, Institute of Accelerator Technologies, Ankara University, Gölbaşı, 06830 Ankara, Turkey; c Cambrium GmbH, Max-Urich-Strasse 3, 13355 Berlin, Germany

**Keywords:** iron–sulfur proteins, Shethna protein I, *Azotobacter vinelandii*

## Abstract

*Azotobacter vinelandii* is a model diazotroph and is the source of most nitrogenase material for structural and biochemical work. *Azotobacter* can grow in above-atmospheric levels of oxygen, despite the sensitivity of nitrogenase activity to oxygen. *Azotobacter* has many iron–sulfur proteins in its genome, which were identified as far back as the 1960s and probably play roles in the complex redox chemistry that *Azotobacter* must maintain when fixing nitrogen. Here, the 2.1 Å resolution crystal structure of the [2Fe–2S] protein I (Shethna protein I) from *A. vinelandii* is presented, revealing a homodimer with the [2Fe–2S] cluster coordinated by the surrounding conserved cysteine residues. It is similar to the structure of the thioredoxin-like [2Fe–2S] protein from *Aquifex aeolicus*, including the positions of the [2Fe–2S] clusters and conserved cysteine residues. The structure of Shethna protein I will provide information for understanding its function in relation to nitrogen fixation and its evolutionary relationships to other ferredoxins.

## Abstract 2

Several *Azotobacter* iron–sulfur proteins probably play roles in the complex redox chemistry that *Azotobacter* must maintain when fixing nitrogen. The 2.1 Å resolution crystal structure of the [2Fe–2S] protein I (Shethna protein I) from *Azotobacter vinelandii* reveals a homodimer similar to the structure of the thioredoxin-like [2Fe–2S] protein from *Aquifex aeolicus*, with the [2Fe–2S] cluster coordinated by the surrounding conserved cysteine residues.

## 1. Introduction   

*Azotobacter vinelandii* is a model organism for nitrogen fixation (Peters *et al.*, 1995 ▸). Although nitrogenase is inactivated by oxygen, *A. vinelandii* can grow in above-atmospheric concentrations of oxygen (Maier & Moshiri, 2000 ▸). This oxygen resistance arises via at least two mechanisms: firstly a high respiratory rate, which removes oxygen, called ‘respiratory protection’ (Jones *et al.*, 1973 ▸), and secondly ‘conformational protection’, in which high oxygen levels pause nitrogenase activity, but on their removal nitrogen fixation resumes. Conformational protection is mediated by [2Fe–2S] protein II (FeSII) or Shethna protein, which forms a complex with nitrogenase that is catalytically inactive but resistant to oxygen (Robson, 1979 ▸; Moshiri *et al.*, 1994 ▸; Schlesier *et al.*, 2016 ▸). The FeSII protein was first purified by Shethna in the 1960s (Shethna *et al.*, 1964 ▸, 1968 ▸) as a nonheme iron protein of interest, together with another protein, the so-called Shethna protein I or [2Fe–2S] protein I (FeSI).

Ferredoxins are iron–sulfur proteins that mediate electron transfer, and there are several families of them. 2Fe ferredoxins can be classified as plant-type, mitochondrial-type and bacterial ferredoxins. There is also another class comprising 3Fe, 4Fe, 7Fe and 8Fe ferredoxins with one or two FeS clusters of the cubane type (Zanetti & Pandini, 2013 ▸). FeSI is a member of the thioredoxin-like ferredoxin family (InterPro family IPR009737). Thioredoxins have no Fe–S clusters, but have pairs of cysteine residues to exchange disulfide-bond oxidation states (Saarinen *et al.*, 1995 ▸). These thioredoxin-like ferredoxins are also present in some multimeric hydrogenases (Appel & Schulz, 1996 ▸; De Luca *et al.*, 1998 ▸) and the NADH-ubiquinone oxidoreductase of respiratory chains (Yano *et al.*, 1994 ▸).

**Figure 1:**
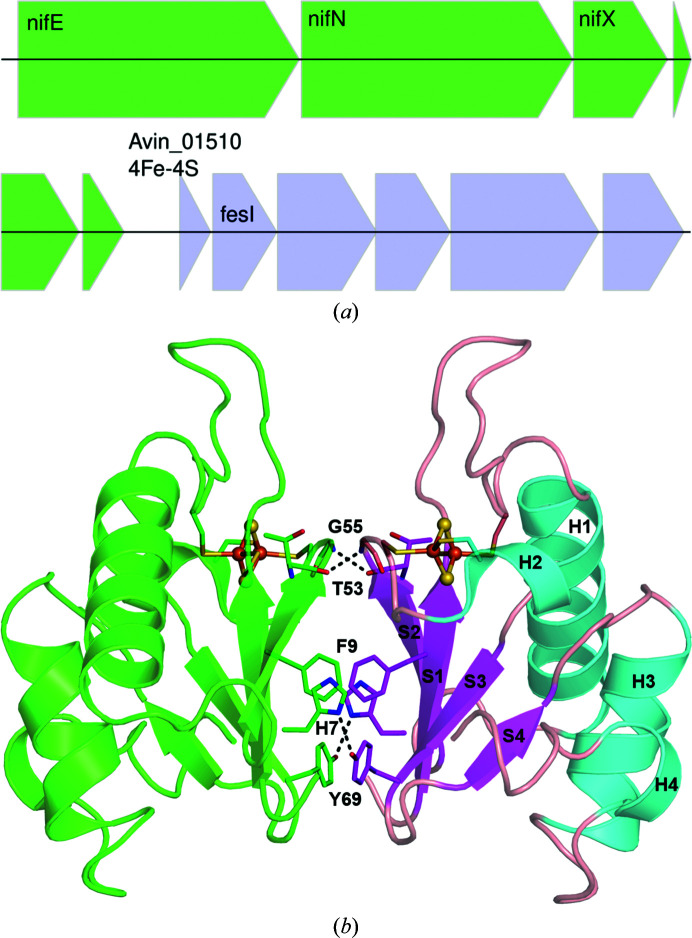
(*a*) Part of the major *nif* region showing the *nifENX* operon (green) and the operon encoding FeSI (blue). Genes of known function are labelled. FeSI is immediately preceded by Avin_01510, a predicted (4Fe–4S) ferredoxin. (*b*) Cartoon view of the *A. vinelandii* FeSI homodimer. Subunits are shown with the [2Fe–2S] clusters and ligating cysteine residues shown as sticks. The right subunit is coloured according to the secondary structure. Strands (S1–S4), helices (H1–H4) and loops are shown in magenta, cyan and salmon, respectively. The residues (His7, Phe9, Thr53, Gly55 and Tyr69) at the dimer interface are shown as sticks. Hydrogen bonds are shown as black dashed lines.

In the *A. vinelandii* genome (Setubal *et al.*, 2009 ▸), several genes encode proteins associated with nitrogen fixation. The dinitrogen reductase in the nitrogenase system is the MoFe protein. The major *nif* cluster of genes contains the nitrogenase structural genes, including those for MoFe and most of the nitrogenase-assembly factors. FeSI is in the middle of the major *nif* cluster, just after the *nifN*, *nifE* and *nifX* genes which encode nitrogenase-assembly factors. FeSI is predicted to be cotranscribed with a [4Fe–4S] ferredoxin-like protein and several other proteins of unknown function (Fig. 1 ▸ *a*). Moreover, the transcript level of FeSI was reported to increase 2.3-fold under nitrogen-fixing conditions (Hamilton *et al.*, 2011 ▸). Therefore, given the genomic location and transcript data, it is likely that FeSI plays some role in nitrogen fixation. FeSI is homologous to the [2Fe–2S] ferredoxin from *Clostridium pasteurianum* (Chatelet & Meyer, 1999 ▸), which interacts with the *C. pasteurianum* nitrogenase MoFe protein (Golinelli *et al.*, 1997 ▸) and is in the *anf* (iron-only) nitrogenase gene cluster of the *C. pasteurianum* genome (Pyne *et al.*, 2014 ▸).

## 2. Materials and methods   

### 2.1. Macromolecule production   

The *fesI* gene (Avin_01520) was amplified by PCR from *A. vinelandii* CA genomic DNA and cloned by Gibson assembly (Gibson, 2011 ▸) into a modified pRSET-A vector with a thrombin-cleavable 6×His tag. The primers (Invitrogen) used for the pRSET-A vector were 5′-GGATCCACGCGGAACCAGACC-3′ (forward) and 5′-GCCCGAAAGGAAGCTGAGTTGGCT-3′ (reverse), and those for the genomic DNA were 5′-GGTCTGGTTCCGCGTGGATCCATGGCCAAACCCGAGTTCCATATC-3′ (forward) and 5′-AGCCAACTCAGCTTCCTTTCGGGCCTACCAGATCTCGGCAGGGGT-3′ (reverse). The FesI-pRSET-A plasmid was transformed into *Escherichia coli* KRX cells. The cells were grown in 1 l Terrific Broth to an OD of 0.6–0.8 and were then induced with 0.1%(*w*/*v*) rhamnose. The cells were grown at 18°C for 18 h after induction, spun at 4000*g* for 15 min, resuspended in 50 m*M* Tris–HCl pH 7.9, 150 m*M* NaCl and disrupted by sonication. Cell debris was removed by centrifugation and filtration. The supernatant was loaded onto a nickel resin affinity column (Generon) and eluted with 500 m*M* imidazole in 50 m*M* Tris–HCl pH 7.9, 150 m*M* NaCl. The His tag was cleaved by adding 50 U thrombin (Sigma) and incubating at 4°C overnight. Finally, the protein was concentrated to ∼15 mg ml^−1^ for crystallization trials. Macromolecule-production information is summarized in Table 1 ▸.

### 2.2. Crystallization   

Thin plate-shaped crystals, which belonged to space group *P*2_1_, were obtained by sitting-drop vapour diffusion using a reservoir solution consisting of 0.1 *M* HEPES pH 7.5, 70%(*v*/*v*) MPD (2,4-methylpentanediol). Crystals were cryoprotected in the mother liquor with ∼30%(*v*/*v*) polyethylene glycol (PEG) 400 and then flash-cooled in liquid nitrogen. Crystallization information is summarized in Table 2 ▸.

### 2.3. Data collection and processing   

X-ray diffraction data were collected from cryoprotected crystals at 100 K on beamline I03 at Diamond Light Source, UK. For phase determination, a data set was collected at the Fe *K*-edge at a wavelength of 1.734 Å. The collected data were processed and scaled with *xia*2 (Winter, 2010 ▸) using the 3dii (*XDS*) setting (Kabsch, 2010 ▸). Data-collection and processing statistics are given in Table 3 ▸.

### 2.4. Structure solution and refinement   

Phases were calculated with the *AUTOSHARP* pipeline (Vonrhein *et al.*, 2007 ▸) and an initial model was built with *ARP*/*wARP* (Langer *et al.*, 2008 ▸). Cycles of model building and refinement with noncrystallographic symmetry restraints were performed in *REFMAC*5 (Murshudov *et al.*, 2011 ▸) and *Coot* (Emsley *et al.*, 2010 ▸). *MolProbity* (Williams *et al.*, 2018 ▸) was used for validation. The final refinement statistics are presented in Table 4 ▸. Figures were drawn with *PyMOL* (DeLano, 2002 ▸).

## 3. Results and discussion   

**Figure 2:**
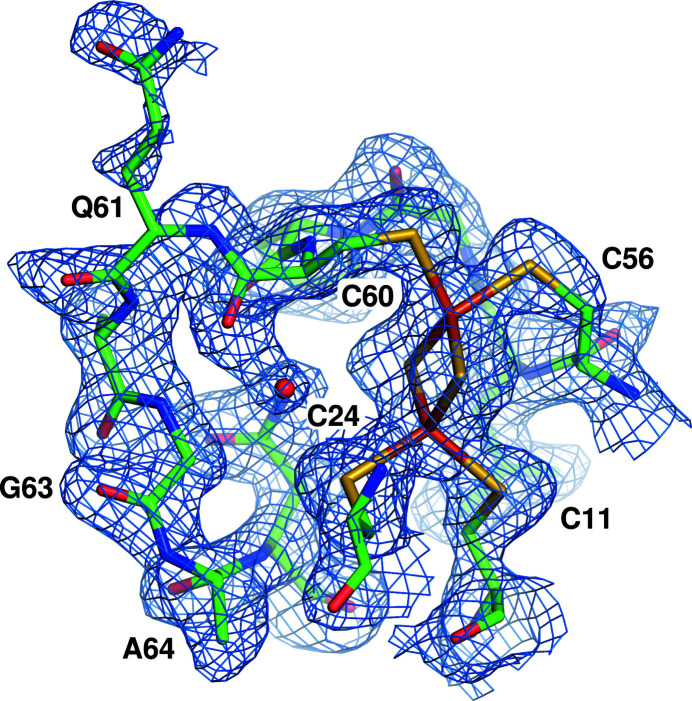
FeSI electron density (2*F*_o_ − *F*_c_) contoured at 1σ around the [2Fe–2S] cluster in chain *A*, with residues of interest labelled. The Gly63–Ala64 bond is a *cis*-peptide.

The *A. vinelandii* FeSI structure is a homodimer burying 1420 Å^2^ as calculated with the *PDBePISA* service at EMBL–EBI (Krissinel & Henrick, 2007 ▸). It consists of a sheet with four β-strands, two long α-helices that pack against the sheet, two short α-helices and several loops. The sheet from the monomer packs against its symmetry mate, forming the dimer interface. On one side of the dimer interface, two hydrogen bonds in parallel strands (S1 and S3) between the side chains of His7 and Tyr69 of two chains and, on the other side, hydrogen bonding between the main chains of Thr53 and Gly55 on strand S2 of two chains, along with the interaction of Phe9 with its symmetry mate through π-stacking, favour the dimerization (Fig. 1 ▸ *b*). The two chains are similar, with an r.m.s.d. of 0.1 Å over 104 C^α^ atoms. Both clusters are coordinated in the same way and were refined at 100% occupancy. The [2Fe–2S] cluster is positioned between two loops that are located between strand S1 and helix H1 and between strand S2 and helix H2. It is bound to four cysteine residues: Cys11, Cys24, Cys56 and Cys60. There is a nonproline *cis*-peptide, well resolved in the density, between Gly63 and Ala64 (Fig. 2 ▸), which are Gly–Pro in many homologous sequences (Supplementary Fig. S1).

The most similar protein to FeSI with a known structure is the [2Fe–2S] ferredoxin from *Aquifex aeolicus* (Chatelet *et al.*, 1999 ▸; Yeh *et al.*, 2000 ▸), with an r.m.s.d. of 2 Å over one chain and 38% sequence identity. *A. aeolicus* does not fix nitrogen, so its thioredoxin-like ferredoxin must have a function that is unrelated to nitrogen fixation.

**Figure 3:**
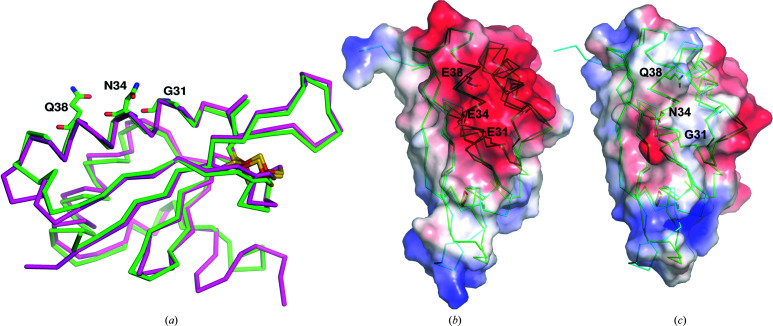
(*a*) Superposition of FeSI from *A. vinelandii* (green) and the [2Fe–2S] ferredoxin from *A. aeolicus* (magenta; PDB entry 1f37). In FeSI, Gly31, Asn34 and Gln38 are shown as sticks. (*b*) Electrostatic surface representation of the predicted model of the *C. pasteurianum* [2Fe–2S] protein and its superposition with FeSI, shown as cyan (*C. pasteurianum* [2Fe–2S] protein) and green (FeSI) ribbons. The key residues (Glu31, Glu34 and Glu38) forming the negatively charged surface are highlighted as sticks. (*c*) Electrostatic surface representation of FeSI and its superposition with the model of the *C. pasteurianum* [2Fe–2S] protein, shown as coloured ribbons. The key residues (Gly31, Asn34 and Gln38) are highlighted as sticks.

FeSI has 31% sequence identity to the *C. pasteurianum* ferredoxin (Chatelet & Meyer, 1999 ▸), which has no experimental structure. The structure of *C. pasteurianum* ferredoxin was predicted in *trRosetta* (Yang *et al.*, 2020 ▸) and the model has an r.m.s.d. of 0.6 Å to FeSI over one chain. *C. pasteurianum* ferredoxin specifically and strongly interacts electrostatically with the nitrogenase protein MoFe via three negatively charged residues: Glu31, Glu34 and Glu38 (Golinelli *et al.*, 1997 ▸). These residues are on an α-helix on the opposite side of the protein to the [2Fe–2S] cluster. In FeSI, the equivalent residues are Gly31, Asn34 and Gln38, all of which are on the outside face of the first helix of the structure (Fig. 3 ▸). The change in charge from three negative glutamates to a neutral glutamine, asparagine and glycine suggests that it is unlikely that FeSI interacts directly with *A. vinelandii* MoFe by this mechanism and that its involvement in nitrogen fixation is of a different nature to the *C. pasteurianum* protein. Moreover, previous cross-linking experiments did not find any interaction of FeSI with the *C. pasteurianum* or *A. vinelandii* MoFe proteins (Chatelet & Meyer, 1999 ▸).

The FeSI structure completes the structural picture of the original two *Azotobacter* FeS proteins purified by Shethna in the 1960s. The structure of the FeSI protein from *A. vinelandii* appears to be a typical thioredoxin-like ferredoxin and will provide information for understanding its function in relation to nitrogen fixation and its evolutionary relationships to other ferredoxins.

## Supplementary Material

PDB reference: [2Fe–2S] protein I from *Azotobacter vinelandii*, 5abr

Sequence alignment of [2Fe-2S] proteins from A. vinelandii, A. aeolicus and C. pasteurianum. DOI: 10.1107/S2053230X21009936/uf5005sup1.pdf

## Figures and Tables

**Table 1:** Macromolecule-production information

Source organism	*A. vinelandii* CA
DNA source	*A. vinelandii* CA genomic DNA
Forward primer (vector)	GGATCCACGCGGAACCAGACC
Reverse primer (vector)	GCCCGAAAGGAAGCTGAGTTGGCT
Forward primer (genomic DNA)	GGTCTGGTTCCGCGTGGATCCATGGCCAAACCCGAGTTCCATATC
Reverse primer (genomic DNA)	AGCCAACTCAGCTTCCTTTCGGGCCTACCAGATCTCGGCAGGGGT
Expression vector	pRSET-A
Expression host	*E. coli* (KRX)
Complete amino-acid sequence of the construct produced	MRGSHHHHHHGLVPRGSMAKPEFHIFICAQNRPAGHPRGSCGAKGAEGVYNAFAQVLIQKNLTNRIALTTTGCLGPCQAGANVLIYPGAVMYSWVEPADAAIIVEQHLLGGEPYADKLTPAEIW

**Table 2:** Crystallization

Method	Sitting-drop vapour diffusion
Plate type	96-well plate
Temperature (K)	290
Protein concentration (mg ml^−1^)	15
Buffer composition of protein solution	50 m*M* Tris–HCl pH 7.9, 150 m*M* NaCl
Composition of reservoir solution	0.1 *M* HEPES pH 7.5, 70%(*v*/*v*) MPD
Volume and ratio of drop	200 nl (1:1) and 300 nl (2:1)
Volume of reservoir (µl)	50

**Table 3:** Data collection and processing Values in parentheses are for the outer shell.

Diffraction source	I03, Diamond Light Source
Wavelength (Å)	1.734
Temperature (K)	100
Detector	PILATUS
Crystal-to-detector distance (mm)	185
Rotation range per image (°)	0.2
Total rotation range (°)	180
Exposure time per image (s)	0.1
Space group	*P*12_1_1
*a*, *b*, *c* (Å)	39.04, 60.10, 45.51
α, β, γ (°)	90, 109.37, 90
Resolution range (Å)	42.93–2.11 (2.16–2.11)
No. of unique reflections	11386 (859)
Completeness (%)	98.7 (97.9)
Multiplicity	3.1 (3.0)
〈*I*/σ(*I*)〉	8.1 (2.4)
Overall *B* factor from Wilson plot (Å^2^)	21.4

**Table 4:** Structure refinement Values in parentheses are for the outer shell.

Resolution range (Å)	42.9–2.1 (2.165–2.110)
Completeness (%)	98.5 (97.9)
No. of reflections, working set	11386 (827)
No. of reflections, test set	545 (29)
Final *R*_cryst_	0.177
Final *R*_free_	0.231
No. of non-H atoms	
Protein	1558
Ligand	8
Solvent	128
Total	1694
R.m.s. deviations	
Bonds (Å)	0.024
Angles (°)	2.373
Average *B* factors (Å^2^)	
Protein	35.6
Ligand	23.4
Water	43.0
Ramachandran plot	
Favoured regions (%)	97.0
Additionally allowed (%)	3.0
