# Supplementary material for: Crystal structure of the [2Fe–2S] protein I (Shethna protein I) from Azotobacter vinelandii
Source: Acta Crystallogr F Struct Biol Commun. 2021 Oct 19;77(Pt 11):407–11. doi: 10.1107/S2053230X21009936 (PMC8561814; doi:10.1107/S2053230X21009936)
Supplement: Supplementary file 1 [file f-77-00407-sup1.pdf]

Acta Cryst F

STRUCTURAL BIOLOGY COMMUNICATIONS

**Volume 77 (2021)**

**Supporting information for article:**

# Crystal structure of the [2Fe–2S] protein I (Shethna protein I) from *Azotobacter vinelandii*

**Burak V. Kabasakal, Charles A. R. Cotton and James W. Murray**

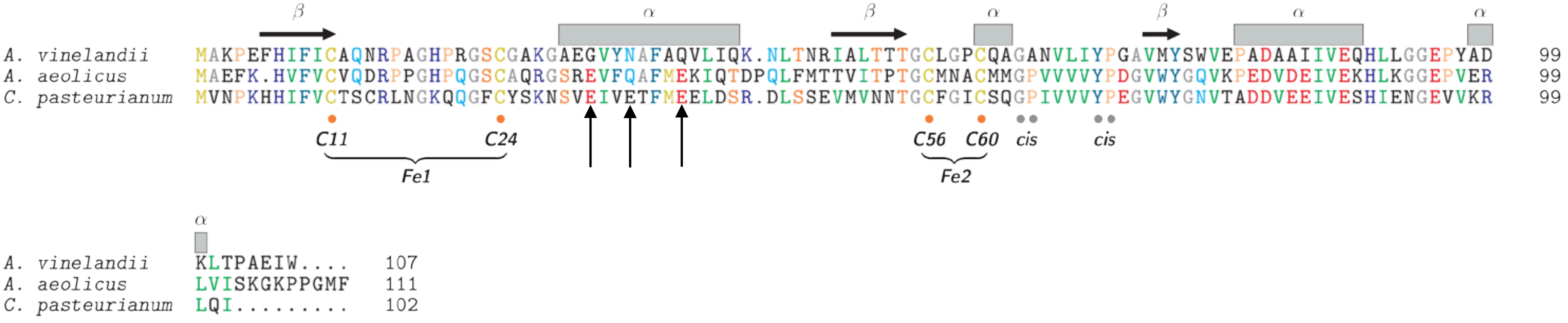


**Supplementary Fig. S1.** Sequence alignment of [2Fe-2S] proteins from *A. vinelandii*, *A. aeolicus*, and *C. pasteurianum*. Conserved cysteines coordinating [2Fe-2S] clusters are highlighted with orange dots, the non-proline cis peptide (between Gly63 and Ala64) in A. vinelandii FeSI, and Gly-Pro cis peptides are highlighted with grey dots. Glu31, Glu34, and Glu38 in *C. pasteurianum* correspond Gly31, Asn34 and Gln38 in *A. vinelandii* FeSI.
